# Counting the cost of not breastfeeding is now easier, but women’s unpaid health care work remains invisible

**DOI:** 10.1093/heapol/czz064

**Published:** 2019-07-11

**Authors:** Julie P Smith

**Affiliations:** Department of Health Services Research and Policy, Research School of Population Health, College of Health and Medicine, Australian National University, Canberra, Australia

**Key Messages**

- The new tool for estimating the country costs of not breastfeeding is an important advance that highlights the extent of women’s invisible economic contribution to national economies and health care systems in caring for infants and young children.
- The tool excludes the costs of additional unpaid household care for sick children, making its cost estimates highly conservative. Ironically, the costing tool entrenches thereby gender bias in economic and health care measurement.
- Such exclusion gives rise to the startling paradox that Norway presents as having comparatively high-economic costs of not breastfeeding.
- Properly accounting for costs of not breastfeeding requires more adequate national time use data collection, and cost analyses that incorporate non-market household production.

In this issue, ‘The Cost of Not Breastfeeding’ (Walters *et al.*, 2019) launches a tool for estimating economic losses from low breastfeeding rates. The study concludes that global economic costs of not breastfeeding are substantial, around US$341 billion annually, but can be addressed by investing resources in key breastfeeding strategies and interventions.

The authors should be congratulated for their wide-scale investigation. Their study advances the important analyses in the 2016 *Lancet Breastfeeding Series* (Rollins *et al.*, 2016) and earlier studies. The pioneering economic study on this topic was in *Health Policy and Planning* over two decades ago (Horton *et al.*, 1996).

Most children are not breastfed as recommended (Victora *et al.*, 2016), with a contemporary worldwide boom in commercial milk formula sales (Baker *et al.*, 2016). International agencies and non government organizations (NGOs) are increasingly aware of the economic significance of this, and of the urgency for adequately resourcing enabling breastfeeding policies (Holla-Bhar *et al.*, 2015; Kakietek *et al.*, 2017; Walters *et al.*, 2017). In 2015, the Vice President of the World Bank (Hansen, 2015, 386) declared that ‘in sheer raw bottom line economic terms, breastfeeding may be the single best investment a country can make’.

‘The Cost of Not Breastfeeding’ fills an important knowledge gap for both global and national health policy-makers. The online tool will facilitate wider application of its methodologies by policy-makers and programme agencies. Most importantly, it will support evidence-based advocacy for investing in policies and programmes to improve infant and young child feeding practices.

## Limitations

### Limitations of the conceptual framework

The ‘cost of not breastfeeding’ tool has an important defect—it does not count women’s unpaid time caring for sick children, time which is therefore treated as ‘free’. This is wrong. Women’s work, paid or unpaid, has an economic opportunity cost. Excluding unpaid work time from costs of not breastfeeding is innately gender biased, and risks further entrenching gender inequity in economic and health care measurement, and policy evaluation. The authors follow previous literature in this regard. Not accounting for unpaid health care means the online tool will replicate women’s invisibility in economic statistics.

The authors were hindered by the dearth of national data on household time use. There are important gaps in national time use surveys (van de Ven and Zwijnenburg, 2018), which would otherwise provide the data for measuring time cost burdens on unpaid health caregivers, and their contribution to health system cost containment. This reflects in part the lack of effective demand from important users of time use data (Collas-Monsod, 2011; van de Ven and Zwijnenburg, 2016; Zwijnenburg, 2017).

The importance of measuring time costs for effective policy and programme design was illustrated long ago for developing countries’ health and nutrition programmes (Leslie, 1989). Omitting opportunity costs of caregiving in evaluating the true costs of health interventions is a crucial and ongoing problem.

*Unpaid* health *and child care provided in the household, along with other activities that contribute to the physical, cognitive, and emotional development of members of a household, have a major impact on individual and public well-being, as well as on the human development potential of the countries. These economic activities, performed largely by women, take place outside the market and are therefore invisible in the economic statistics and national accounts systems of most countries* [Pan American Health Organization (PAHO), 2010].

Measuring the time costs of caring for sick non-breastfed infants would make clear that adequate access to paid maternity leave is a key intervention to increase breastfeeding. Breastfeeding is not free; it too has a time cost (Smith and Forrester, 2013).

This draws attention to the broader gender bias within current economic statistical systems. Time spent by members of the household on non-market productive activities (including activities such as breastfeeding) is invisible in economic statistical systems governed by the System of National Accounts (Commission of the European Communities IMF, 1993). When measured, the economic value of unpaid household production including care work is the equivalent of 15–70% of gross domestic product (GDP) across a variety of countries (van de Ven and Zwijnenburg, 2018).

This has important consequences.

*Actually, the invisibility of the unpaid work of women in delivering health services to other members of the household and the community, and in developing the human capital of new generations, prevents a proper analysis of the impact of public policies on them and hinders a definition of broader economic and social development strategies. Few countries have fiscal adjustment or sectoral financing policies that explicitly consider the impact of changes in the quantity and quality of in-home health service delivery as a result of such policies, nor do many countries have social and economic development strategies and policies in place that acknowledge the importance of unpaid work in the household* [Pan American Health Organization (PAHO), 2010, p. vii].

### Limitations of the epidemiological evidence

The authors foreshadow the need to update the tool; its morbidity and mortality estimates currently exclude relevant conditions such as childhood diabetes, sudden infant death syndrome and necrotizing enterocolitis (Smith and Harvey, 2011; Victora *et al.*, 2016). This highlights that a ‘cost of not breastfeeding’ approach can only go so far in evaluating economic gains from improved infant and young child feeding practices. Breastfeeding is an evolved mammalian behaviour of wide ranging importance to human health and development (Volk and Atkinson, 2013; Sellen, 2016). Hence, such estimates will continue to be limited by contemporary scientific knowledge on consequences of exposing human babies to an early diet of processed cows’ milk (Smith, 1999).

Norway is an interesting case study. Norway counts its human milk supply in national food statistics (Norwegian Health Directorate, 2017). Norwegian researchers pioneered valuing human milk using market values (Oshaug and Botten, 1994). Using this approach the market value of ‘lost’ production of human milk in Norway due to not breastfeeding at recommended levels has been estimated at around $600 million a year (Smith, 2013). The ‘Cost of Not Breastfeeding’ tool is limited by large gaps in relevant data for morbidity and mortality, and child development in Norway.

A macro-economic framework perhaps suggests a way forward, and reinforces the need for time use data collection. The economic value of breastfeeding within country food production systems has been recognized since at least the early 1970s (Berg, 1973), and analysed within a national accounting framework (Smith and Ingham, 2005; Smith, 2018). Leading economic experts now acknowledge that human milk should be counted in GDP, but is not. As observed by the influential French Presidential Commission on Measurement of Economic Performance and Social Progress (Stiglitz *et al.*, 2009a);

*There is a serious omission in the valuation of home-produced goods – the value of breast milk. This is clearly within the System of National Accounts production boundary, is quantitatively non-trivial and also has important implications for public policy and child and maternal health* (Stiglitz *et al.*, 2009b).

## Concluding remarks

The ‘Cost of Not Breastfeeding’ study provides an important contribution to the literature, and a tool to inform and advance policy-making in this area, but it is far from capturing the full economic implications of low breastfeeding rates. It is a lost opportunity to address the gender bias inherent when women’s time counts for nothing in economic analysis tools. The new tool risks perpetuating the invisibility of the substantial economic contributions and costs incurred by women engaged in unpaid care work through breastfeeding or caring for infants and young children. Such invisibility distorts economic policy-making, biases government budget funding priorities, and hinders the realization of women’s and children’s human rights to health, nutrition and economic equality [Himmelweit, 2002; Pan American Health Organization (PAHO), 2010; Balakrishnan *et al.*, 2011]. Valuing women’s time would, for example highlight the importance of paid maternity leave to reduce the costs of breastfeeding as recommended.

Future tool development in this field needs to consider the unpaid work of caregivers. Rapid growth in the number of time use surveys including in LMICs, points to the feasibility and usefulness of collecting this type of data [Charmes, 2015; Folbre, 2018; International Labour Office (ILO), 2018].

Perhaps it is time for OECD Statistics to prioritize work with agencies such as WHO, the World Bank and NGOs such as Alive & Thrive on addressing this important global health and nutrition issue.

## Funding

JPS received funding from the Australian Research Council under a Future Fellowship (FT140101260). The funding body had no role in the design or conduct of the research.

*Conflict of interest statement.* None declared. 
